# Designing Peptide-Based HIV Vaccine for Chinese

**DOI:** 10.1155/2014/272950

**Published:** 2014-07-06

**Authors:** Jiayi Shu, Xiaojuan Fan, Jie Ping, Xia Jin, Pei Hao

**Affiliations:** ^1^Viral Disease and Vaccine Translational Research Unit, Institut Pasteur of Shanghai, Chinese Academy of Sciences, Room 507, Building B, Life Science Research Building, 320 Yueyang Road, Shanghai 200031, China; ^2^Vaccine Centre, Institut Pasteur of Shanghai, Chinese Academy of Sciences, 320 Yueyang Road, Shanghai 200031, China; ^3^Bioinformatics Platform, Institut Pasteur of Shanghai, Chinese Academy of Sciences, Room 405, Building B, Life Science Research Building, 320 Yueyang Road, Shanghai 200031, China

## Abstract

CD4+ T cells are central to the induction and maintenance of CD8+ T cell and antibody-producing B cell responses, and the latter are essential for the protection against disease in subjects with HIV infection. How to elicit HIV-specific CD4+ T cell responses in a given population using vaccines is one of the major areas of current HIV vaccine research. To design vaccine that targets specifically Chinese, we assembled a database that is comprised of sequences from 821 Chinese HIV isolates and 46 human leukocyte antigen (HLA) DR alleles identified in Chinese population. We then predicted 20 potential HIV epitopes using bioinformatics approaches. The combination of these 20 epitopes has a theoretical coverage of 98.1% of the population for both the prevalent HIV genotypes and also Chinese HLA-DR types. We suggest that testing this vaccine experimentally will facilitate the development of a CD4+ T cell vaccine especially catered for Chinese.

## 1. Introduction

Over 30 million people have died from HIV/AIDS related illnesses since HIV was discovered in the 1980s. There are currently 33 million of HIV carriers [1]. The rate of new infection is still on the rise globally. In China, HIV infection is a great concern, especially in southern part of China, for example, Yunnan, Sichuan, Guangxi, and Xinjiang Provinces, where a large number of infected people are drug users. Additionally, in the regions of Henan, Hubei Provinces where people were infected through illicit blood collection, the rate of infection reached up to 60% of blood donors [2]. Highly active antiretroviral therapy (HAART), a combination of three or more antiretroviral drugs, is routinely used to treat individuals with HIV infection [3]. It significantly extends the lifespan and improves the quality of life of people infected with HIV but cannot eradicate the virus [4]. The course of treatment is life-long and the medicines are expensive. In developing countries, available antiretroviral drugs are still limited. Therefore, a preventive HIV vaccine is especially needed.

HIV genome is comprised of nine structural (*Env*,* Gag,* and* Pol*) and regulatory (*Tat*,* Rev*,* Nef*,* Vif*,* Vpr,* and* Vpu*) genes. The* pol* gene encodes for reverse transcriptase which is error prone. This leads to high mutation rate, 15–20% divergence between the nucleic acid sequences of different HIV clades, and 7–12% variability within each clade [5]. Although the base composition of HIV genome is stable [6], host immune response further increases the HIV nucleotide diversity.

Due to the extreme sequence diversity and high mutation rate of HIV, it has been difficult to develop an efficacious HIV vaccine. A successful HIV vaccine requires inducing neutralizing antibodies and cytotoxic T cell responses, both of which can only be optimally induced and maintained in the presence of a concurrent CD4+ T helper cell response [7]. Despite many years of basic and clinical research, to date, there are only three major human HIV vaccine clinical trials completed. Set up in 1998, AIDSVAX gp120 protein vaccine is the first HIV vaccine going through Phase III trial in human and targeted to induce neutralizing antibody activity. Although antibodies to homologous virus were elicited, they failed to neutralize heterologous viruses [8]. In 2004, a Phase IIb trial with Merck's MRKAd5, which is a trivalent vaccine including* gag*,* pol*, and* nef* genes in an adenovirus 5 vector, is designed for inducing cytotoxic T cell responses [9, 10]. Despite the induction of significant level of IFN gamma-producing T cells, the MRKAd5 has increased the risk of HIV acquisition in vaccine recipients and failed to reduce viral load after HIV infection [11]. Later in 2009, a Phase III trial of RV144 HIV-1 vaccine was completed in Thailand, which is a vaccine combination comprised of ALVAC (a vaccine containing genetically engineered versions of* gag*,* env*, and* pol* inserted in canarypox vector) and AIDSVAX (a bivalent gp120 envelope protein vaccine). These vaccines are theoretically capable of eliciting both CD8+ T cell response and neutralizing antibody response. Despite neither vaccine worked alone, in the combination, they unexpectedly lowered the HIV incidence by 31.2% in vaccine recipients; however, they did not reduce viral load [12]. These large clinical trials have opened new questions and revealed new opportunities for HIV vaccine research, including a rethinking of the need for a vaccine for CD4+ T helper cells.

In order to stimulate a CD4+ T helper cell response, antigens need to be processed and presented through MHC class II molecules. The form of antigen could be either whole protein or peptide epitopes. A previous study with a subunit vaccine comprised of 18 CD4+ T helper cell epitopes has demonstrated an efficient induction of robust helper T cell response in a Phase I clinical trial in Caucasian population [13]. Whether a similar strategy works in Chinese population requires to be tested.

To select antigenic epitopes for a vaccine, one must address several issues. One, HIV exhibits high mutation rates, and thus conserved sequences may be needed to cover a given population. Two, the human leukocyte antigen (HLA) is highly polymorphic, and it restricts the proportion of individuals who will respond to a particular antigen [14, 15]. To overcome these problems, promising T cell epitopes that bind to several HLA alleles for maximal population coverage should be selected [16], and a large variety of HIV sequences should be considered in the design of a HIV vaccine.

MHC class II is a heterodimer that is comprised of a monomorphic *α* and a highly polymorphic *β* chain. There are over 400 class II alleles identified, spreading among HLA-DM, HLA-DO, HLA-DP, HLA-DQ, and HLA-DR loci. Among them DRB1 is the most polymorphic gene, consists of 221 alleles; followed by DPB1 and DQB1 that has 84 and 39 alleles, respectively. Whereas other gene loci may have only 1 or 2 alleles [17]. Therefore, DRB1 is the best choice to optimize MHC II coverage. The frequency of HLA-DRB1 serotype differs among ethnic groups. Within DRB1 allotype, DRB1∗11 and 13 serotypes present in 16% and 14% of black population, whereas, in Caucasoid and Chinese, DRB1∗07 and DRB1∗11 and DRB1∗12 and DRB1∗15 appear in the highest percentage [17]. The above evidences support the development of a new HIV vaccine specifically for Chinese population. Such a vaccine should have higher probability in dealing with circulating HIV serotypes in China.

To overcome these complex issues of vaccine design, bioinformatics methods may help to determine common features of vaccine antigens that have potential to deal with divergent population and HIV quasispecies. Specifically, bioinformatics-based approach is the most feasible method in screening a large set of peptide epitopes and selection of promising vaccine antigens. In this study, we extracted 821 HIV sequence and 46 Chinese DRB1 alleles from public information and compiled a database. A combination of 7 public available epitope prediction algorithms was used to screen the database and identify CD4+ T cell epitopes as HIV vaccine antigens. We selected a set of 20 epitopes, which in combination could cover more than 98% of our target population.

## 2. Materials and Methods

### 2.1. Data Collection and Methods for Epitope Prediction

In total, 821 HIV whole genome sequences of Chinese population were retrieved from HIV Database (http://www.hiv.lanl.gov/) [27], and the distribution of 46 HLA-DR alleles (Table 1) was extracted from The Allele Frequency Net Database (AFND) (http://www.allelefrequencies.net/) [28].

Seven existing methods available in Immune Epitope Database (IEDB) [29] for MHC class II binding were used to predict HIV epitopes based on binding affinity between HLA DR types and HIV epitopes. These methods included Consensus method [30], NN-align (netMHCII-2.2) [31], stabilization matrix alignment method (SMM-align) [32], Sturniolo [33], average relative binding (ARB) [34], NetMHCIIpan [35], and Combinatorial library (ComLib) [30].

### 2.2. Epitope Selection

All epitopes are 15 amino acids in length. To be a potential epitope, it must have a MHC binding affinity threshold of IC_50_ = 500 nM or below. A selected epitope was removed from the epitope pool before the next prediction. The process is repeated until all epitopes were selected. All calculations of epitope selection process were conducted in INFORSENSE Knowledge Discovery Environment (KDS) software platform [36]. The mathematical model used to calculate the predictive score for each DR allele of known coverage (as listed in Table 1) is the following equations:

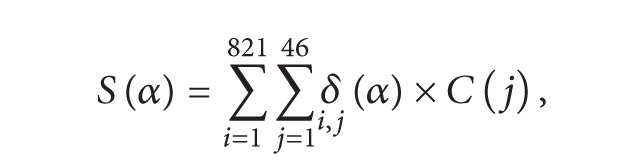
(I)

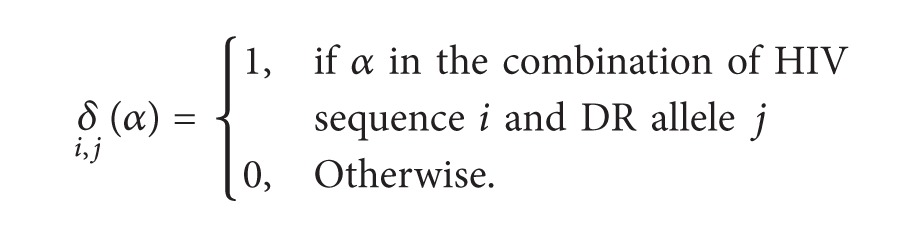
(II)


In the first equation *(I)*, *α* represents the epitope; *C*(*j*) is the percentage coverage of number *j* DRB1 allele; *δ*(*α*) is the function to indicate whether the epitope exists in the combination of HIV sequence and DR allele, existence scored 1, and absence scored 0. *S*(*α*) is the sum of number of times of the binding of HIV sequence *i* and DR allele *j* after being standardized to the proportion of DR allele *j* in all DRB1 alleles. All DRB1 alleles included in the study cover 95.2% all Chinese HLA-DR alleles [28].

**Figure 1:**
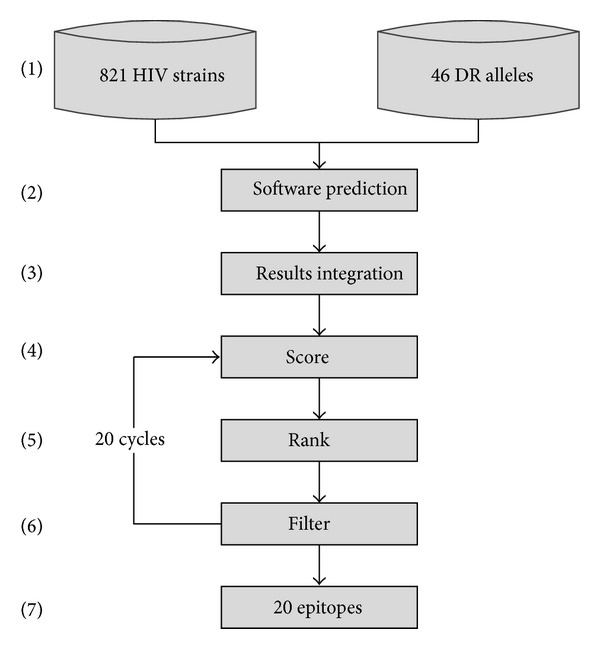
A flowchart illustrates procedures for CD4+T cell epitope prediction. (1) Using KDS platform with datasets of 821 circulating HIV-1 strains and 46 HLA-DRB1 alleles in Chinese population; (2) the software predicted possible epitopes by 7 known methods from the IEDB database; (3) all results were combined and scored using *(I)* and *(II)*; (4) the epitopes were ranked according to the score; (5) the epitope with the top score and the lowest IC_50_ value was selected; (6) the selected epitope was then removed from the epitope pool; (7) steps 4–6 were repeated until all 20 epitopes fulfilled the criteria that were selected.

We selected epitopes from a combined pool of epitopes through KDS platform using 7 prediction methods from IEDB with a dataset that consisted of 821 circulating HIV genome sequences in China and 46 Chinese HLA-DRB1 alleles. The epitopes bind to MHC class I molecules that were removed first, and then the value of IC_50_ was considered. Next, we ranked all epitopes based on the coverage score (the higher the better coverage in HIV genome and DR-HLA alleles). After an epitope has been selected, it was removed from the database before next selection. This process was repeated until 20 epitopes were selected. The workflow diagram of this procedure was illustrated in Figure 1.

## 3. Results

### 3.1. The Coverage Distribution of HLA-DR of Chinese Population

A total of 46 HLA-DR alleles were identified from AFND (Table 1). The alleles were listed and its coverage in Chinese population was given. The table showed the coverage ranged from 0.1% (DRB1∗08:09) to 6.77% (DRB1∗07:01) and in a total of 95.2% of the Chinese population. The sample population comprises 1704 individuals of the Han ethnicity. This information was obtained from ten regions within the mainland, China, and two other regions, Hong Kong and Singapore, where Chinese ethnicity dominates. Among them, the DRB1-02, -05, and -06 genotypes were not detected.

### 3.2. The Diversity of Epitope Coverage

**Figure 2:**
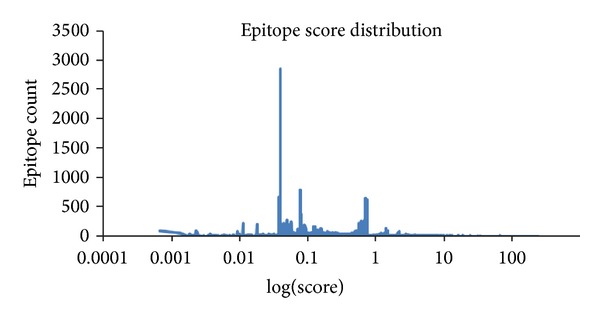
The distribution of epitope coverage score. The epitope coverage scores (log-transformed) were plotted on the horizontal axis against the frequency of epitope count on the vertical axis. Most of the log score localized to the region between 0.01 and 1.

With a combination of 7 existing epitope prediction methods in IEDB, using database comprised of 46 different DRB1 alleles and 821 full genome sequences of HIV isolates circulating in China, we then predicted 38,460,402 potential epitopes. After duplicates were removed, 21,007,527 potential epitopes remained. We scored these epitopes based on the allele coverage and total coverage score, which was in general normally distributed. As shown in Figure 2, most epitopes displayed low coverage scores, 0.1 or lower; the highest epitope count reached approximately 3000.

### 3.3. HIV Epitopes Specifically for Chinese Population

By using the methods described above, we obtained 20 epitopes, which in theory covered all 46 DRB1 allelic genotypes and 821 Chinese HIV sequences (Table 2). All 20 epitopes were selected for binding to MHC class II and absence of binding to MHC class I. Table 2 listed the amino acid sequences of the 20 epitopes, their location in HIV-1 gene, their percentage of coverage in HIV-1 genome sequences from 4% to 43%, the proportion in the HLA-DR allele sequences between 52% and 100%, and the total coverage in both sequences as low as 4% and the highest at 41%. One single epitope WIILGLNKIVRMYSP covered 41% of both DRB1 and Chinese specific HIV-1 genome sequences, which is of note. This epitope had been reported before [18]. In fact, 4 other predicted epitopes (LNKIVRMYSPTSILD, GFPVRPQVPLRPMTY, VDRFYKTLRAEQASQ, and LYKYKVVKIEPLGVA) have also been published previously [22–24, 26] and 4 peptide sequences (PVVSTQLLLNGSLAE, LRIIFAVLSIVNRVR, ILDLWVYHTQGYFPD, and YKRWIILGLNKIVRM) were reported in patents before [19–21, 25], whereas the remaining 11 epitopes have never been reported. All 20 epitopes together provided 98.1% coverage in HIV genome and HLA-DR alleles. These predicted epitopes were found in HIV-1* gag*,* env*,* pol*, and* nef* genes. Six of them were in* gag* gene, 6 in* env*, 2 in* pol*, and 6 in* nef*.

We then applied the new method to a previously published HIV vaccine comprised of T helper epitopes and tested in clinical trial [13]. The table listed 17 epitopes, from* gag*,* pol*,* env,* and* vpu* genes. One published epitope that has a HIA binding IC_50_ above our threshold of 500, Env 566 IKQFINMWQEVKAMY, was not listed. For these epitopes, HIV coverage is from 2% to 43%, DR coverage is between 35% and 98%, and specific coverage is at highest of 41% and in sum of 69%.

## 4. Discussion

In this paper, we described a novel method for designing a peptide-based T helper cell vaccine for HIV, which is specific for Chinese HIV strains and Chinese MHC class II genotypes. The current method has several advantages. First, our methodology of epitope prediction is easily accessible to public use. In fact, it is a combination of all seven existing methods publically available in IEDB. The IEDB database comprises a series of most up-to-date and evidence based methods specifically created for the prediction of MHC restricted T cell epitopes. In contrast to other studies that only used one of the methods, we used them all for more accurate prediction of MHC class II restricted T helper epitopes.

So far, there are three major types of bioinformatics methods for the prediction of MHC class II restricted T helper cell epitopes. One is called matrix alignment algorithm, and these are SMM, ARB, and Sturniolo methods. This algorithm uses published T cell epitopes and their respective binding affinity to MHC class II, in terms of the IC_50_ value, to determine epitopes. The other relies on machine learning, and NN-align and NetMHCIIpan methods belong to this category. New sequences are subjected to computer simulated models to predict whether any epitopes can bind to a particular MHC II to high enough affinity. The third type combines several methods together to predict epitopes. These include Consensus method and ComLib method.

Consensus method was reported to provide highest true positive rate, followed by NN-align and ARB [37]. NetMHCIIpan performed the best among all other pan-specific methods for MHC class II with varied experimental settings [38]. NN-align performs especially well in handling large dataset among all other machine learning methods and in combination with ARB outperforms the use of NN-align alone [30].

In this study, we used all above seven methods simultaneously, scored the potential epitopes independently, and then used IC_50_ value as a filter to select T cell epitopes that have the broadest population coverage. Our method did not use all 8 IEDB recommended methods but integrated 7 of the IEDB methods because the 8th IEDB method is an integration of the other seven and thus not an independent measurement. The method we used could be considered as “greedy” algorithm in the bioinformatics field, which predicts the best epitope among all in a pool of potential epitopes. Thus, we believe an integrated method that uses a combination of all seven original algorithms might be the best to predict more accurately MHC class II epitopes.

Another unique feature of our study is that we designed candidate helper T cell vaccine targets specifically to the Chinese population. Most common world circulating HIV subtypes are B and C, and recombinant forms are AE and AG. In contrast, the common subtypes are B and recombinant forms are BC and AE in China [27, 39, 40]. We extracted all 821 subtypes of HIV-1 strains which are mostly subtypes B and C for developing a highly specific vaccine for Chinese population. As T helper cell epitopes are recognized through MHC class II, and that Chinese exhibit divergence DRB1 alleles, we also included 46 published Chinese HLA-DRB1 genotypes into our prediction.

In comparison to a previous paper that selected MHC class II binders according to the binding affinity to multiple HLA-DR subtypes [13], we focused on DRB1 alleles which are most polymorphic among human MHC class II loci and thus directed our study to be more specific and increased possibility to induce T cell responses specifically for Chinese.

One limitation in our study, as shown in Table 1, is that DRB1 genotypes 2, 5, and 6 were not included. This is due to a lack of publication of any information on DRB1∗02, 05, and 06. Therefore, our dataset represents what is currently available; that is, there are only 46 DRB1 alleles in Chinese population.

By using our method, we obtained 20 helper T cell epitopes which covered 98.1% of HIV strains known to have been circulating in China and all Chinese HLA-DR genotypes. There are limited studies that have tested designed peptide T helper vaccine in humans. In a published paper that contains 18 T helper epitopes [13], our combination of epitope predication methods found that these epitopes covered 69% of Chinese HIV genomes (Table 3). In a different population that is predominantly Caucasian, these epitopes combined have a 100% coverage. Thus, the difference in the coverage may suggest our predicting method is more specific for Chinese population, and our epitopes are better potential HIV vaccine candidate for Chinese. Furthermore, 9 epitopes we obtained have been published before and 11 are not. Thus, we both have the empirical evidence to support that our allelic specific peptides have the potential to stimulate T cell responses and new epitopes to suggest that our prediction is innovative.

There was one core epitope WIILGLNKIVRMY, appeared in both studies, showing very high HIV, HLA-DR, and specific coverage. The Gag epitope with two amino acids modification WIILGLNKIVRMYSP was reported to stimulate strong CD4+ T responses [27]; another variant of the same epitope KRWIILGLNKIVRMY exhibited superior HLA-DR binding capacity [13]. Another difference between our study and that published is that our epitopes consisted of those in* nef* gene but not* vpu* gene, whereas Walker's study did not cover* nef* but* vpu*. These comparisons suggest that a vaccine designed predominantly for Caucasian may not be optimal for Chinese population. One epitope, for instance, Env 566 (IKQFINMWQEVKAMY) [13], given in Walker's paper, was not picked up in our study.

Our method predicted epitopes, in theory, together covered 98.1% of HIV-1 genome and Chinese specific DRB1 alleles. In comparison, Walker's study reported 18 T helper cell epitopes that cover 100% of the global population. By using a prediction algorithm which based mostly on HLA supertypes [13]. However, when submitted to our new prediction method, the same epitopes only achieved 69% of coverage of the Chinese population. The discrepancy in methods for prediction may give different results. Further experimental evidence is required to find out whether our method is more accurate.

The allele coverage of DRB1 for Chinese was based on 1704 subjects of whom 1569 were from mainland China and 135 were from Hong Kong and Singapore. All Chinese allele data regarding DRB1 frequencies were extracted from AFND, and all 1704 subjects were Chinese Han ethnics. There is no information on other minor national groups in China available. This may lead to inaccuracy in prediction of helper T cell epitopes for the Chinese. Larger sample size may improve the quality of our prediction.

## 5. Conclusions

In this study, we report a novel bioinformatics method for designing peptide epitope based T helper vaccine for HIV. We suggest further in vitro and in vivo experiments to be performed to test the immunogenicity of this vaccine and improvement of method of prediction to be made when necessary.

## Figures and Tables

**Table 1:** The DRB1 allele coverage in Chinese population.

Number	Alleles	Coverage
1	DRB1∗01:01	0.0145
2	DRB1∗01:02	0.0014
3	DRB1∗03:01	0.0514
4	DRB1∗03:07	0.0009
5	DRB1∗04:01	0.0120
6	DRB1∗04:02	0.0024
7	DRB1∗04:03	0.0238
8	DRB1∗04:04	0.0082
9	DRB1∗04:05	0.0413
10	DRB1∗04:06	0.0233
11	DRB1∗04:07	0.0041
12	DRB1∗04:08	0.0075
13	DRB1∗04:10	0.0030
14	DRB1∗04:17	0.0018
15	DRB1∗07:01	0.0677
16	DRB1∗08:01	0.0018
17	DRB1∗08:02	0.0076
18	DRB1∗08:03	0.0512
19	DRB1∗08:04	0.0029
20	DRB1∗08:09	0.001
21	DRB1∗08:12	0.0011
22	DRB1∗09:01	0.0490
23	DRB1∗10:01	0.0149
24	DRB1∗11:01	0.0669
25	DRB1∗11:03	0.0015
26	DRB1∗11:04	0.0154
27	DRB1∗11:06	0.0013
28	DRB1∗12:01	0.0518
29	DRB1∗12:02	0.1048
30	DRB1∗13:01	0.0227
31	DRB1∗13:02	0.0233
32	DRB1∗13:03	0.0029
33	DRB1∗13:12	0.0025
34	DRB1∗14:01	0.0214
35	DRB1∗14:02	0.0013
36	DRB1∗14:03	0.0091
37	DRB1∗14:04	0.0078
38	DRB1∗14:05	0.0193
39	DRB1∗14:07	0.0023
40	DRB1∗15:01	0.1139
41	DRB1∗15:02	0.0418
42	DRB1∗15:04	0.0013
43	DRB1∗15:05	0.0018
44	DRB1∗16:01	0.0029
45	DRB1∗16:02	0.0401
46	DRB1∗16:05	0.0032

Total		0.9520

**Table 2:** Predicted HIV T helper cell epitopes for Chinese population.

Amino acid sequences	Protein destination^1^	HIV%^2^	DR%^3^	Total coverage^4^	Specific coverage^5^	Reference^6^
WIILGLNKIVRMYSP	Gag 265	43%	89%	41%	40.72%	Younes et al., 2003 [18]
PVVSTQLLLNGSLAE	Env 262	38%	74%	34%	27.73%	August et al., 2013 [19]
VQMAVFIHNFKRKGG	Pol 892	24%	93%	23%	9.79%	NA (IEDB)
LRIIFAVLSIVNRVR	Env 702	24%	96%	26%	4.39%	Sette et al., 2005 [20]
ILDLWVYHTQGYFPD	Nef 127	12%	63%	10%	5.37%	Sette et al., 2002 [21]
LNKIVRMYSPTSILD	Gag 284	25%	100%	25%	1.81%	Korber et al., 2001 [22]
WGIKQLQARVLAVER	Env 588	22%	87%	20%	1.25%	NA
GAFDLSFFLKEKGGL	Nef 91	4%	63%	4%	1.20%	NA
VDRFYKTLRAEQATQ	Gag 297T	15%	98%	15%	1.17%	NA
GFPVRPQVPLRPMTY	Nef 85	9%	65%	6%	0.84%	Korber et al., 2002 [23]
TPGIRYQYNVLPQGW	Pol 295	23%	93%	22%	0.78%	NA
VDRFYKTLRAEQASQ	Gag 297S	15%	98%	15%	0.71%	Bozzacco et al., 2012 [24]
RQLLSGIVQQQSNLL	Env 549	27%	83%	26%	0.56%	NA
GLIYSKKRQEILDLW	Nef 117	6%	67%	5%	0.50%	NA
KPCVKLTPLCVTLNC	Env 126	17%	89%	16%	0.28%	NA
YKRWIILGLNKIVRM	Gag 272	43%	89%	41%	0.24%	Sette et al., 2002 [25]
PLTFGWCFKLVPVDP	Nef 144	11%	52%	11%	0.21%	NA
FGWCFKLVPVDPREV	Nef 147	4%	93%	4%	0.24%	NA
CKQIIKQLQPALQTG	Gag 67	8%	98%	8%	0.16%	NA
LYKYKVVKIEPLGVA	Env 489	6%	100%	6%	0.14%	Dzuris et al., 2001 [26]

Total specific coverage^7^					98.1%	

^1^The location of epitopes on HIV viral gene products and the first amino acid of the viral gene product.

^2^The epitope sequence presented in the proportion of 821 HIV genome sequences.

^3^The epitope sequence presented in the proportion of 46 DR alleles.

^4^The ratio of the epitope appeared in both 821 HIV genome and DR allele sequences.

^5^Calculated based on the coverage of the epitope in the rest of the dataset after removing the preceding epitope.

^6^Reference where the epitope had been published. NA: not available in published literature.

^7^Sum of specific coverage for all 20 epitopes.

**Table 3:** Using novel algorism to calculate the coverage of epitopes in a published T helper vaccine for Chinese population.

Amino acid sequence	Protein destination^1^	HIV%^2^	DR%^3^	Specific coverage^4^
FRKYTAFTIPSINNE	Pol 303	14%	98%	13%
EKVYLAWVPAHKGIG	Pol 711	3%	98%	3%
GEIYKRWIILGLNKI	Gag 294	20%	87%	18%
KRWIILGLNKIVRMY	Gag 298	43%	89%	41%
GAVVIQDNSDIKVVP	Pol 989	21%	57%	12%
YRKILRQRKIDRLID	Vpu 31	2%	89%	2%
QKQITKIQNFRVYYR	Pol 956	19%	98%	19%
SPAIFQSSMTKILEP	Pol 335	11%	93%	11%
QHLLQLTVWGIKQLQ	Env 729	23%	83%	21%
AETFYVDGAANRETK	Pol 619	7%	41%	2%
QGQMVHQAISPRTLN	Gag 171	3%	85%	3%
WAGIKQEFGIPYNPQ	Pol 874	3%	35%	1%
KVYLAWVPAHKGIGG	Pol 712	3%	93%	2%
KTAVQMAVFIHNFKR	Pol 915	24%	83%	22%
EVNIVTDSQYALGII	Pol 674	24%	57%	16%
WEFVNTPPLVKLWYQ	Pol 596	22%	91%	22%
HSNWRAMASDFNLPP	Pol 758	11%	57%	7%

Total specific coverage^5^				69%

^1^The epitopes were selected from a published paper.

Data in columns 2–5 were calculated using the same method as in Table 2.
